# Psychological symptoms during and after Austrian first lockdown in individuals with bipolar disorder? A follow-up control-group investigation

**DOI:** 10.1186/s40345-021-00222-8

**Published:** 2021-06-01

**Authors:** Nina Dalkner, Jolana Wagner-Skacel, Michaela Ratzenhofer, Frederike Fellendorf, Melanie Lenger, Alexander Maget, Adelina Tmava-Berisha, René Pilz, Robert Queissner, Carlo Hamm, Susanne Bengesser, Martina Platzer, Armin Birner, Eva Reininghaus

**Affiliations:** 1 Department of Psychiatry and Psychotherapeutic Medicine, Medical University Graz, Auenbruggerplatz 31, Graz, 8036 Austria; 2 Department of Medical Psychology and Psychotherapy, Medical University of Graz, Auenbruggerplatz 3, 8036 Graz, Austria

**Keywords:** Bipolar disorder, COVID-19, Lockdown, Global symptom load, Somatization, Depression, Anxiety

## Abstract

**Background:**

The coronavirus disease (COVID-19) pandemic, a global health crisis, has resulted in widespread socioeconomic restrictions including lockdown, social distancing, and self-isolation. To date, little is known about the psychological impact of the COVID-19 pandemic and lockdown on patients with bipolar disorder as a particularly vulnerable group.

**Methods:**

An online survey was conducted in Austria at two points of measurement (T1 April 2020 during the first lockdown vs. T2 May 2020 at post-lockdown). The sample comprises 20 patients with bipolar disorder (mean age = 49.4 ± 15.6 years) and 20 healthy controls (mean age = 32.7 ± 9.6 years). A 2 × 2 factorial design to compare two time points (T1 vs. T2) and two groups (patients vs. healthy controls) was used. Main outcome measures included the Brief Symptom Inventory-18 (BSI-18) and a (non-validated and non-standardized) assessment to determine COVID-19 fears and emotional distress due to social distancing. Multiple linear regression analyses were used to assess the longitudinal association of COVID-19 fears/emotional distress due to social distancing during lockdown (T1) and psychological symptoms after lockdown (T2).

**Results:**

At T1, results demonstrated higher scores in BSI-18 subscales depression, anxiety and global severity index as well as emotional distress due to social distancing in bipolar patients compared to controls. There was a significant time x group interaction in the BSI-18 subscale somatization showing a decreasing trend in patients with BD compared to controls. No time effects in BSI-18 subscales or COVID-19 fears/emotional distress due to social distancing were observed. Regression analyses showed that COVID-19 fears during lockdown predicted somatization, only in patients.

**Conclusions:**

There was a connection between the lockdown measures and somatization symptoms observed in patients. When the first steps of easing the social restrictions in May 2020 took place, somatization decreased only in the bipolar compared to the control group. Higher COVID-19 fears during lockdown predicted later symptoms at post-lockdown. Long-term impacts of the COVID-19 pandemic need further investigations to improve current therapeutic approaches and prevent fears and distress during lockdown in individuals with bipolar disorder in times of crisis.

## Introduction

The severe acute respiratory syndrome coronavirus-2 (SARS-CoV-2), which can cause the novel coronavirus disease (COVID-19) and its consequences (e.g., quarantine, lockdown, and social distancing), represent a global health crisis, and the whole world has experienced a state of emergency (Guan et al. 2020. To date, in October 2020, the ongoing COVID-19 pandemic has infected more than 33 million people and led to more than one million deaths globally (Johns Hopkins University 2020). The COVID-19 pandemic is leading directly or indirectly to extraordinary challenges for mental health services (Fatke et al. 2020; Rajkumar 2020). Insecurity, confusion, emotional isolation, and stigma may affect mental health and well-being (Pfefferbaum and North 2020). Psychological stress symptoms (including depression, somatization, insomnia, and anxiety) can be consequences of such an extraordinary situation, including feelings of threat and uncertainty (Lieberman et al. 2020; Pérez-Fuentes et al. 2020). The effects may lead to severe emotional reactions, unhealthy behaviours, or noncompliance with public health directives (Pfefferbaum and North 2020). Furthermore, emotional responses to the COVID-19 pandemic can result in relapse or worsening of an already existing psychiatric disorder because of high susceptibility to stress compared with the general population (Pérez-Fuentes et al. 2020).

BD is a recurrent chronic psychiatric disorder characterized by fluctuations in mood state and energy (Vieta et al. 2016), and can cause people to have a more reactive response to stress when compared to healthy controls. Studies show that individuals with BD exhibit a high risk of developing severe affective episodes during periods with high chronic stress (Bender and Alloy 2011; Malkoff-Schwartz et al. 2000; Weiss et al. 2015). The vulnerability-stress model postulates a neurobiological, polygenic genetic predisposition in concatenation with chronic stress and acute triggers (e.g., critical life events, disruption of the sleep–wake rhythm), which determine the course of psychiatric disorders by gene-environment interactions (e.g., infections and inflammation) mediated by epigenetic modifications, gene expression changes, and other cellular mechanisms (Bengesser and Reininghaus 2018; Brietzke et al. 2012; Esterwood and Saeed 2020). The COVID-19 crisis may be a life event in itself. In this paper, we are especially interested whether patients with BD are more susceptible to psychological symptoms in an extreme environmental situation, as it was the case during lockdown.

Since the outbreak of the COVID-19 pandemic, numerous studies evaluating psychiatric symptoms in individuals with severe mental illness including samples with BD have been published (Chang et al. 2020; Costa et al. 2020; Frank et al. 2020; Hao et al. 2020; Iasevoli et al. 2020; Pan et al. 2020; Riblet et al. 2020; Solé et al. 2021; Zou et al. 2020). Rates of high perceived stress severity, anxiety, and severe depressive symptoms were significantly higher in psychiatric patients compared with controls (Frank et al. 2020; Iasevoli et al. 2020, Solé et al. 2021). In addition, patients reported on sleeping problems (Frank et al. 2020) and fatigue (Zou et al. 2020). A very recent study from Australia, focusing on psychosocial distress in patients with mood disorders, highlighted the maladaptive situational and lifestyle changes during the COVID-19 pandemic of this patient group. In particular, patients with BD reported high stress levels and men with BD had even higher levels of depression than women with BD in this study. Respondents with BD were more concerned about financial issues associated with COVID-19 compared to those with depressive disorder and those with no mental disorder. However, mood disorders in this study were self-reported (Van Rheenen et al. 2020). A further reason for psychological stress symptoms caused by the COVID-19 pandemic might be social distancing rules that were promulgated and enforced by many governments to prevent an uncontrolled spread of SARS-CoV-2.

Lockdown and social distancing (also known as physical distancing) included in infection control actions is intended to slow the spread of disease by minimizing close contact between individuals. However, lockdown, quarantine, and social distancing procedures may lead to loss of personal freedom, uncertainty, fear of the future, and financial well-being, and they also may contribute strongly to widespread emotional distress and mental health problems (Pfefferbaum and North 2020; Semo and Frissa 2020; Zandifar and Badrfam 2020). An Italian study by Rossi et al. (2020) identified associations of lockdown measures with high rates of depression, anxiety, insomnia, perceived stress symptoms and adjustment disorder symptoms in the general population. Brooks et al. (2020) reviewed the psychological impact of quarantine and found negative psychological effects, including post-traumatic stress symptoms, confusion, and anger. Stressors included longer quarantine duration, infection fears, frustration, boredom, inadequate supplies, inadequate information, financial loss, and stigma. Accordingly, Tull et al. (2020) found that being under a stay-at-home order was associated with greater health anxiety, financial worry, and loneliness in healthy adults.

In psychiatric samples, lockdown measures including temporary shutdown of medical and mental health treatment and the disrupted rhythm of a healthy life, such as reduced opportunities to exercise, experience sunlight exposure, and participate in meaningful activities, could pose a special risk or relapse during the pandemic (Muruganandam et al. 2020; Youngstrom et al. 2020). Hao et al. (2020) confirmed that patients with psychiatric disorders experienced more psychiatric symptoms during the COVID-19 pandemic. They highlighted the severity of negative psychological impact on psychiatric patients during strict lockdown measures. An Italian study by Carmassi et al. (2020), surveying 100 patients with BD in April 2020, found more post-traumatic stress symptoms, more anxiety, and depressive symptoms during the period of national lockdown and ongoing social distancing measures. Post-traumatic stress symptoms were related to work and financial difficulties. Interestingly, acute manic symptoms seemed to be protective in this study. A Spanish study by González-Blanco et al. (2020) evaluated depression, anxiety, and stress symptoms as early responses to the pandemic and found that patients with severe mental disorders (bipolar and psychotic disorders) reacted to the lockdown restrictions with higher anxiety levels compared to healthy controls. Solé et al. (2021) stated that lockdown had a higher psychological impact in psychiatric patients vs. controls and that psychiatric patients used less adaptive copings strategies to face the lockdown. Moreover, suicidality case reports as well as increased suicidality rates related to the COVID-19 pandemic because of fear and xenophobia have already been reported (Dsouza et al. 2020; Mamun and Griffiths 2020; Sher 2020). Consequently, several adjustment strategies to prevent psychological stress and suicide attempts during the COVID-19 pandemic have already been published (Ho et al. 2020; Klomek 2020; Wu et al. 2020). Interestingly, there are also few studies showing decreased psychiatric symptoms due to the pandemic. A longitudinal study by Orhan et al. (2020) observed less psychiatric symptoms in older patients with BD (age > 50 years) during the course of the COVID-19 pandemic than at baseline. Psychiatric symptoms were associated with loneliness, not having children, more feelings of loneliness, lower mastery, passive coping style and neuroticism.

Yao et al. (2020) pointed out that individuals with psychiatric disorders are generally more susceptible to infections for multiple reasons including cognitive impairment and little awareness of risk, more comorbidities and more barriers in accessing timely health services. Accordingly, it is suggested that patients with BD exhibit more serious COVID-19-related symptoms (Stefana et al. 2020) and that they are at increased infection risks due higher prevalence of obesity, cardiovascular disease, diabetes mellitus, and obstructive pulmonary disease (Staudt et al. 2019; McIntyre et al. 2007; Vancampfort et al. 2013; Vancampfort et al. 2016; Zareifopoulos et al. 2018). There are already recommendations for patients with BD for the time of the COVID-19 pandemic to decrease the vulnerability, such as proactively improving social connections, prioritizing self-care, and learning to use mobile and telehealth effectively (Stefana et al. 2020; Youngstrom et al. 2020). However, more multidisciplinary research on COVID-19 effects on severe symptoms of BD is needed to accomplish a consequent establishment of prevention strategies for psychiatric COVID-19 consequences (Courtet et al 2020; Holmes et al. 2020).

This study aimed at investigating the psychosocial strain of Austrian patients with BD during the COVID-19 pandemic at two time points (April 2020 vs. May 2020) via an online survey. Austria was in the first lockdown from March 15 to the end of April 2020, and the survey was conducted twice: April 9–April 28, 2020 (T1) and the second time from May 5–June 4, 2020 (T2). Individual differences in the emotional response to the pandemic and effects of governmental restrictions including social distancing and lockdown might be critical for the comprehension of psychological pandemic effects and the construction of new management and treatment models. Thus, we conducted a single-institution prospective analysis to address the following research questions:

1. Do patients with BD and healthy controls differ in a) global severity index, anxiety, depression, and somatization in the course of the pandemic (during and post-lockdown) and in b) COVID-19 fears and emotional distress due to social distancing?
2. Do COVID-19 fears and emotional distress due to social distancing during lockdown (T1) can predict psychological symptoms post-lockdown (T2) in patients vs. healthy controls?

We hypothesized that psychological symptoms in individuals with BD depend on lockdown measures and that patients with BD may report more psychological stress symptoms (anxiety, depression, somatization) during the first Austrian lockdown (vs. post-lockdown) in comparison to a healthy control group. In addition, we assumed that COVID-19 fears and emotional response to social distancing will be related to psychological symptoms, resulting in higher effects in patients.

## Materials and methods

### Study design

Data collection (T1) via online survey began on April 9 and ended on April 28, 2020. Between May 5 and June 4, 2020, the survey was open for answering for a second time (T2) for the same participants. The first survey in April was during the first total lockdown, starting at March 16, 2020 with governmental measures to limit the spread of COVID-19 in Austria. At this time all nonessential shops, schools, nurseries, and leisure premises were closed, all events were cancelled, and home-office rather than in-office work was recommended (except for key workers) or workers were put on furlough. Essential venues were only accessible when wearing face masks and keeping a distance of at least one meter. Furthermore, the public was advised to stay at home and to limit all real-life contacts except for people sharing the same household. Travel to and from Austria was heavily restricted by the government. Up to April 9, 13,120 cases were confirmed in Austria, of which 7585 people were actively infected and 295 people had died in connection with COVID-19; by April 28, 15,357 cases were confirmed, of which 2208 were actively infected and 569 had died (Nussmayr 2020).

**Fig.1:**
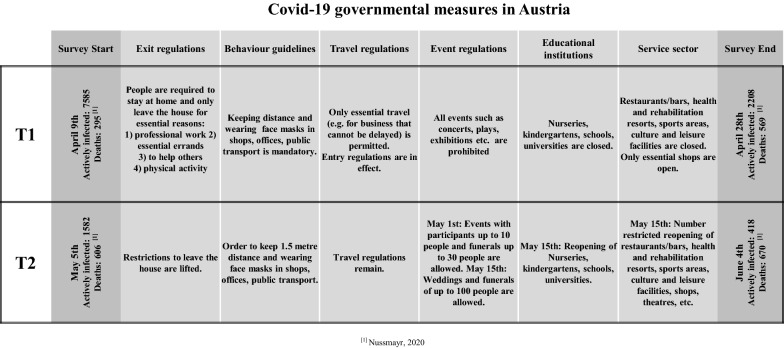
Overview of the COVID-19 protective measures in Austria in April 2020 (T1) and May 2020 (T2)

The follow-up survey at T2 was conducted when restrictions where loosened, including permission for events with up to 10 people, and all shops, leisure venues, and hairdressers were allowed to open again. In the middle of May, number-restricted reopening of schools, restaurants, and places of worship took place. Wearing a face mask inside and keeping distance was still mandatory, and travel restrictions remained. At the end of May, events up to 100 people were allowed again. Up to May 5, 15,650 cases were confirmed in Austria, of which 1,582 people were actively infected and 606 people had died in connection with COVID-19; by June 4, 16,805 cases were confirmed, of which 418 were actively infected and 670 had died in Austria (Nussmayr 2020). An overview of the governmental measures in Austria at both time points of this study is given in Fig. 1.

### Participants

This study surveyed former participants of the BIPLONG study, and all patients were diagnosed earlier with the Structured Clinical Interview for DSM-IV (SCID-I) and treated at the outpatient center for BD at the Medical University of Graz, Department of Psychiatry and Psychotherapeutic Medicine. Participants answered the questionnaires pseudo-anonymously using a participant code of former studies (see our recent report on the BIPLONG study by Dalkner et al. 2020). Inclusion criteria included voluntary participation (informed consent), German native speakers, age above 18 years, and e-mail access. We surveyed 38 patients with BD and 39 healthy controls at T1 and 23 patients with BD and 22 healthy controls at T2. Total data sets at both time points including all relevant variables were available from 20 participants with BD vs. 20 healthy controls. The healthy controls included were also former study participants and had no psychiatric disease or positive family history of a psychiatric disease. This was screened in the BIPLONG study and again in the COVID-19 survey. This study was conducted in accordance with the Declaration of Helsinki and was approved by the Ethics Committee of the Medical University Graz (EK-number: 25-335 ex 12/13). Participants could withdraw from the survey at any moment without providing any justification.

### Psychological inventories

The web-based survey, in German, was accessible online using the survey tool LimeSurvey; it solicited answers to the following question topics:

Global symptom load was assessed using the German version of the Brief Symptom Inventory-18 (BSI-18; Franke et al. 2017) by Derogatis and Fitzpatrick (2004), a short version of the Symptom-Checklist-90 Revised (Derogatis and Unger 2010). The BSI-18 comprises 18 items assessing psychological distress in the last 7 days on three subscales (depression, anxiety, and somatization). The global severity index (GSI) is a global measure of psychological distress. The BSI-18 employs a 5-point rating form ranging from 1 (“absolutely not”) to 5 (“very strong”). The subscales (total value of each scale is 24) show an internal consistency with a Cronbach’s alpha of α = 0.82 for Somatization, α = 0.87 for Depression, α = 0.84 for Anxiety and α = 0.93 for GSI. (Derogatis and Fitzpatrick 2004).

The Beck Depression Inventory (BDI-2) was used as a self-evaluation report to assess the severity of depressive symptoms, with 21 items and a 4-point Likert scoring system. The total score ranges from 0 to 63 points. According to the manual, a score below 18 indicates a lack of clinical depression. The scale has been shown to demonstrate an internal consistency with a Cronbach’s alpha of α ≥ 0.84 and a reliability of *r* ≥ 0.75 (Kühner et al. 2007).

The Altman Self-Rating Mania Scale (ASRM) by Altman et al. (1997) is a five-item scale for assessing mood, self-confidence, sleep disturbances, speech, and activity level during 1 week. Each question can be rated with 0 to 4 points, and a total score above five indicates mania.

Demographic information included personal data (sex, age, education, relationship status), information about the living situation (inhabitants in home town, people in household, children), engagement in activities and hobbies, the presence of a daily structure, adherence to medication (changes in current medication) and governmental measures, and contact with a mental health professional (psychiatrist, psychologist, psychotherapist).

A COVID-19 assessment was developed by the Department of Psychiatry and Psychotherapeutic Medicine at the Medical University of Graz assessing COVID-19 fears, as follows (0 = no fears, 10 = extremely high fear):

- On a scale from 0 to 10, how strongly do you rate your concerns and fears about the coronavirus?
- On a scale from 0 to 10, how strongly do you rate your fear of contracting the coronavirus?
- On a scale from 0 to 10, how strongly do you rate your fear of infecting others with the coronavirus?

The three items showed a highly significant intercorrelation (all *p* < 0.01), and a mean index for COVID-19 fears was created, showing a Cronbach’s alpha of α = 0.81 at T1 and α = 0.82 at T2.

Emotional distress due to social distancing was assessed by the following five items on a 6-point rating scale (0 = not at all, 4 = full commitment):

- On a scale from 0 to 4, social distancing makes me feel lonely/bored/frustrated/hopeless/anxious.

Out of these significantly intercorrelated items (all *p* < .01), a mean index for “Emotional distress due to social distancing” was created, showing a Cronbach’s alpha of α = .90 at T1 and α = .86 at T2.

### Statistical analyses

A 2 × 2 factorial design to compare two time points (T1 vs. T2) × 2 groups (patients with BD vs. healthy controls) was used. Consequently, repeated measures ANCOVAs (controlling for age, sex, education, and employment) for analyzing global symptom load and subscales of the BSI-18 from same subjects at both time points (combining data from April vs. May) were calculated. For analyses of variance, effect sizes are presented as Cohen’s partial eta-squared (η^2^). Paired *t*-tests were post-hoc computed to demonstrate changes in psychological scales from T1 to T2 within one group. Multiple linear regression analyses controlling for age, sex, education, and employment were applied to assess whether COVID-19 fears as well as emotional distress due to social distancing during lockdown (T1) as independent variables can predict psychological symptoms (somatization, depression, anxiety, GSI) after lockdown (T2); multiple regression models were compared between groups. Basic statistical requirements to run multiple regression analyses were met and preliminary analyses were conducted to check relevant assumptions of regression analysis including linearity, normality, absence of multicollinearity, and homoscedasticity. Cohen’s *f2* were calculated as a measure of effect size interpreted according to Cohen (1988) as small (*f2* = 0.02), medium (*f2* = 0.15), and big effects (*f2* = 0.35). Differences in clinical data between patients with BD and healthy controls at one time point were calculated with chi-square tests (for nominal) and the Freeman–Halton Fisher’s exact test, when the expected cell size was < 5, and *t*-tests (for metric data) and Mann–Whitney *U* tests (for nonparametric data). The overall significance was set at 0.05 and all analyses were performed using the Statistical Package for Social Sciences (SPSS version 25.0, IBM).

## Results

### Sample description

The sociodemographic characteristics of participants are shown in Table 1. Fifty percent of the 20 patients and 75.0% of the 20 controls were females. The mean age of the participants was 49.35 ± 15.55 years in the patient group and 32.65 ± 9.58 years in the control group. Patients were more frequently retired (*χ*^*2*^(6) = 22.01, *p* < 0.001) and had a lower educational level (*χ*^*2*^(5) = 10.28, *p* = 0.050; see Table 1). Of all participants, no one had tested positive for COVID-19, no one lived together with someone who tested positive for COVID-19, and no one was put under quarantine (because of contact with positive COVID-19 cases) at the time of testing or before. Patients suffered significantly more from cardiovascular disease than controls (*χ*^*2*^(1) = 5.71, *p* = 0.017). No differences between patients and controls were found in history of diabetes (*χ*^*2*^(1) = 3.24, *p* = 0.231), obstructive pulmonary disease (*χ*^*2*^(1) = 2.11, *p* = 0.487), and hypertension (*χ*^*2*^(1) = 2.11, *p* = 0.487; see Table 2). Of the patients, 25% reported using online counseling or online therapy during the pandemic; however, 30% stated not having any contact with their treating psychiatrist or psychologist during the lockdown. In this study, no sex differences in COVID-19 fears, emotional distress due to social distancing, or psychological symptoms were observed (all *p* < 0.05). The clinical data (somatic comorbidities, medication, and clinical self-ratings with BDI-2 and ASRM) are presented in Table 2.

**Table 1:** Demographic data

	BD (*n* = 20)	Controls (*n* = 20)	Statistics
Sex			
Male (*N*)	10	5	
Female (*N*)	10	15	χ^*2*^*(1)* = 2.67, *p* = 0.102
Age			
Years (*M*, *SD*)	49.35 (15.55)	32.65 (9.58)	*t(38)* = 4.09, *p* < 0.001
Highest education			
Compulsory education (*N*)	2	0	*χ*^*2*^*(5)* = 10.28, *p* = 0.050^a^
Apprenticeship (*N*)	7	1	
A-Levels (*N*)	5	4	
University Bachelor’s Degree (*N*)	1	4	
University Master’s Degree (*N*)	4	7	
University Doctorate (*N*)	1	4	
Marital status			
Married, in relationship (*N*)	12	12	*χ*^*2*^*(3)* = 5.33, *p* = 0.149^a^
Single (*N*)	4	8	
Divorced/Widowed (*N*)	3	0	
Employment			
Unemployed, before the pandemic	1	0	*χ*^*2*^*(6)* = 22.01, *p* < 0.001^a^
Unemployed, due to the pandemic	1	0	
Short-time work	2	1	
Retired/rehabilitation	10	0	
Student	1	3	
Home office	5	10	
Employed	0	6	
Living situation			
Alone (*N*)	5	3	*χ*^*2*^*(6)* = 4.99, *p* = 0.605^a^
With partner (*N*)	5	7	
With partner and children (*N*)	5	3	
With child/children (*N*)	0	1	
In a shared residence (*N*)	1	2	
With parents (*N*)	2	4	
In a multi-generation household (*N*)	2	0	
Inhabitants town of residence			
< 1000 Inhabitants (*N*)	3	1	*χ*^*2*^*(3)* = 6.27, *p* = 0.108
1000–4999 Inhabitants (*N*)	6	1	
5000–9999 Inhabitants (*N*)	2	3	
≥ 10.000 Inhabitants (*N*)	9	15	

*BD*   bipolar disorder

^a^Fisher’s exact test

**Table 2:** Clinical data

	BD (*n* = 20)	Controls (*n* = 20)	Statistics
Mood stabilizers			
Lithium	30%	–	
Atypical antipsychotics	35%	–	
Anticonvulsants	10%	–	
Others	25%	–	
Somatic comorbidities			
Cardiovascular disease	5	0	*χ*^*2*^(1) = 5.71, *p* = 0.017^a^
Diabetes mellitus	3	0	*χ*^*2*^(1) = 3.24, *p* = 0.231^a^
Obstructive pulmonary disease	2	0	*χ*^*2*^(1) = 2.11, *p* = 0.487^a^
Hypertension	6	2	*χ*^*2*^(1) = 2.50, *p* = 0.114
Symptom self-ratings			
BDI-2 (*M, SD*), April	15.45 (10.74)	3.10 (2.53)	*t*(38) = 5.00, *p* < 0.001
BDI-2 (*M, SD*), May	12.20 (11.69)	2.40 (2.21)	*t*(38) = 3.69, *p* < 0.01
ASRM (*M, SD*), April	0.95 (2.28)	0.55 (1.19)	*t*(38) = 0.70, *p* = 0.491
ASRM (*M, SD*), May	1.5 (2.19)	0.25 (0.55)	*t*(38) = 2.48, *p* = 0.022

*BD*  bipolar disorder, *BDI-2*  beck depression inventory-2, *ASRM* Altman self-rating mania scale, *M*  mean, SD  standard deviation

^a^Fisher’s exact test

In April, 90% (vs. 95% in May) of the patients reported taking their medication regularly, and as prescribed without arbitrary change of dosage. In addition, 45% (n = 9) of patients reported subjective negative effects of the COVID-19 pandemic on their mental well-being (vs. 40% of controls; *χ*^*2*^(1) = 1.63, *p* = 0.202), and 25% (n = 5) (vs. 20% of controls; *χ*^*2*^(1) = 0.224, *p* = 0.638) reported positive effects; the rest reported no effect. For a negative valuation of lockdown, patients listed amongst others little social contacts, loss of daily structure, and suspension of psychotherapy. Positive ratings of lockdown comprised amongst others more time for oneself.

### Psychological symptoms at the peak of the lockdown in April 2020 vs. post-lockdown in may 2020

**Fig. 2:**
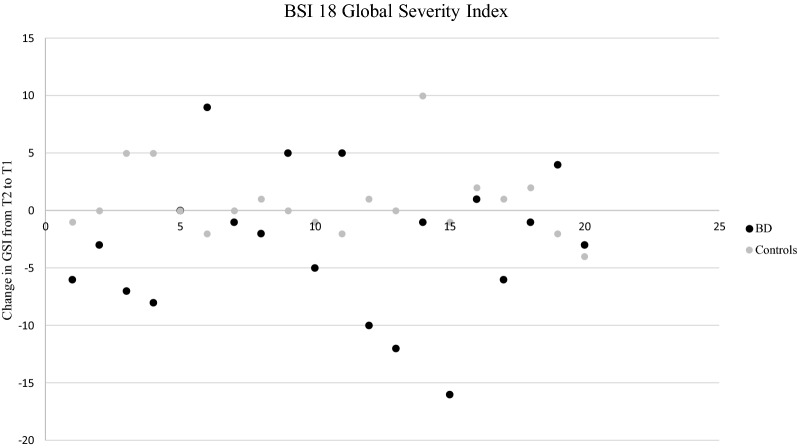
Median change from T1 to T2 in BSI-18 global severity index(GSI) *BD* bipolar disorder, *GSI* global severity index

Patients with BD differed from healthy controls in the median GSI decrease from T1 to T2 (χ^2^(2) = 6.63, *p* = 0.036). Fourteen patients (vs. six healthy controls) showed a decrease and five patients (vs. eight controls) showed an increase in GSI from T1 to T2, with a minimum of − 16 and a maximum of + 9 points in the BD group and − 4 to + 10 points in healthy controls. The other participants showed no difference in GSI from T1 to T2. Figure 2 displays the median change from T1 to T2 in GSI.

## Somatization

**Fig. 3:**
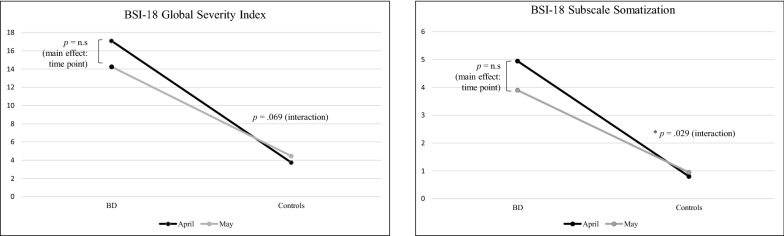
Main result of the two-tailed repeated measure ANCOVAs for BSI-18 somatization and BSI-18 global severity index (GSI)

For the subscale somatization, a two-tailed repeated measures ANCOVA (controlled for age, sex, education, and employment) showed no main effect time point (*F*_(1, 34)_ = 1.20, *p* = 0.280, η^2^ = 0.034) and no main effect group (at T1) (*F*_(1, 34)_ = 2.82, *p* = 0.102, η^2^ = 0.077). As shown in Fig. 3, there was a significant interaction between time point (April vs. May) and group (patients vs. controls) in somatization (*F*_(1, 34)_ = 5.22, *p* = 0.029, η^2^ = 0.133). Post-hoc paired *t*-tests showed a decreasing trend in BD patients (*T*_(19)_ = 1.98, *p* = 0.062), but no change in somatization in healthy controls (*T*_(19)_ = -0.72, *p* = 0.481). The covariables age (*η*^*2*^ = 0.045), sex (*η*^*2*^ = 0.006), education (*η*^*2*^ = 0.002), and employment (*η*^*2*^ = 0.006) showed no significant effects on somatization (all *p* > 0.05). The statistical results, means and standard deviations are presented in Table 3.

**Table 3:** Results from repeated measures ANCOVAs: Psychological symptoms (BSI-18) in patients with BD vs. healthy controls in April (T1) and May 2020 (T2)

	BD (*n* = 20)		Controls (*n* = 20)		Statistics					
	T1	T2	T1	T2	Group effect		Time effect		Time x group interaction	
	*M (SD)*	*M (SD)*	*M (SD)*	*M (SD)*	*F*	*p*	*F*	*p*	*F*	*p*
Somatization	4.95 (5.56)	3.90 (5.43)	0.80 (1.58)	0.95 (1.57)	2.82	0.102	1.2	0.28	5.22	0.029
Depression	7.15 (6.42)	5.50 (6.00)	1.55 (1.90)	1.80 (2.12)	11.73	0.002	0.21	0.651	0.38	0.544
Anxiety	5.00 (4.46)	4.85 (5.37)	1.40 (1.19)	1.70 (2.13)	9.48	0.004	1.3	0.263	1.4	0.245
GSI	17.10 (13.83)	14.25 (14.15)	3.75 (3.42)	4.45 (4.55)	10.08	0.003	0.8	0.378	3.53	0.069

Results from repeated measures ANCOVAs controlling for age, sex, education, and employment

*BD*  bipolar disorder, *BSI-18*   brief symptom inventory-18, *GSI*  global severity index, *M* mean, *SD*  standard deviation

## Depression

For the subscale depression, there was no main effect time point (*F*_(1, 34)_ = 0.21, *p* = 0.651, η^2^ = 0.006), but a main effect group (*F*_(1, 34)_ = 11.73, *p* = 0.002, η^2^ = 0.256) showing higher scores in the patients group compared to controls. No interaction time x group for the BSI-18 subscale depression (*F*_(1, 34)_ = 0.376, *p* = 0.544, η^2^ = 0.011) was found (see Table 3). The covariables age (*η*^*2*^ = 0.022), sex (*η*^*2*^ = 0.017), and education (*η*^*2*^ = 0.073) showed no significant effects on depression (all *p* > 0.05). There was a significant time x employment effect (*F*_(1, 34)_ = 5.08, *p* = 0.031, *η*^*2*^ = 0.130).

## Anxiety

For the BSI-18 subscale anxiety, there was no main effect time (*F*_(1, 34)_ = 1.30, *p* = 0.263, η^2^ = 0.037). A significant group effect indicating higher anxiety scores (*F*_(1, 34)_ = 9.48, *p* = 0.004, η^2^ = 0.218) at T1 in the patient compared to the control group was found. No interaction effect time x group in anxiety was found ((*F*_(1, 34)_ = 1.40, *p* = 0.245, η^2^ = 0.040; see Table 3). The covariables age (*η*^*2*^ = 0.001), sex (*η*^*2*^ = 0.108), education (*η*^*2*^ = 0.003), and employment (*η*^*2*^ = 0.003) showed no significant effects on anxiety (all *p* > 0.05).

## Global severity index

In GSI, we observed a decreasing trend from April to May in patients (see Fig. 3), however, the time x group interaction was not significant (*F*_(1, 34)_ = 3.53, *p* = 0.069, *η*^*2*^ = 0.094). At T1, there was a significant group effect indicating higher GSI scores in patients compared with healthy controls (*F*_(1, 34)_ = 10.08, *p* = 0.003, η^2^ = 0.229). Time *(η*^*2*^ = 0.023), age (*η*^*2*^ = 0.047), sex (*η*^*2*^ = 0.001), education (*η*^*2*^ = 0.021), and employment (*η*^*2*^ = 0.034) showed no significant effects on GSI (all *p* > 0.05). Table 3 shows the means and standard deviations as well as ANCOVA statistics of BSI-18 scales.

## COVID-19 fears and emotional distress due to social distancing

Two-tailed repeated measures ANCOVAs for COVID-19 fears and emotional distress due to social distancing showed no significant time effects in COVID-19 fears (*F*_(1, 34)_ = 2.73, *p* = 0.108, η^2^ = 0.074) or emotional distress due to social distancing (*F*_(1, 34)_ = 0.44, *p* = 0.511, η^2^ = 0.013). At T1, there was a significant group difference in emotional distress due to social distancing indicating more distress in patients compared with controls (*F*_(1, 34)_ = 6.62, *p* = 0.015, η^2^ = 0.163), no group difference was found in COVID-19 fears (*F*_(1, 34)_ = 0.84, *p* = 0.366, η^2^ = 0.024). There were no significant time x group interactions in COVID-19 fears (*F*_(1, 34)_ = 0.21, *p* = 0.651, η^2^ = 0.006) or emotional distress due to social distancing (*F*_(1, 34)_ = 0.003, *p* = 0.958, η^2^ = 0.000; see Table 4. Age (*η*^*2*^ = 0.027), sex (*η*^*2*^ = 0.004), education (*η*^*2*^ = 0.019), and employment (*η*^*2*^ = 0.054) showed no significant effects on COVID-19 fears (all *p* > 0.05) and on emotional distress due to social distancing (age: *η*^*2*^ = 0.007; sex: *η*^*2*^ = 0.015; education: *η*^*2*^ = 0.050, employment: *η*^*2*^ = 0.028). The means and standard deviations of the COVID-19 fear index and emotional distress due to social distancing index are shown in Table 4.

**Table 4:** Results from repeated measures ANCOVAs: COVID-19 fears and emotional distress due to social distancing in patients with BD vs. healthy controls in April (T1) and May 2020 (T2)

	BD (*n = *20)		Controls (*n = *20)		Statistics					
	T1	T2	T1	T2	Group effect		Time effect		Time x group interaction	
	*M (SD)*	*M (SD)*	*M (SD)*	*M (SD)*	*F*	*p*	*F*	*p*	*F*	*p*
COVID-19 fears	4.43 (2.69)	3.98 (2.39)	3.79 (2.15)	3.42 (2.14)	0.83	0.366	2.73	0.108	0.21	0.651
ED-SD	1.56 (1.16)	1.13 (1.04)	0.78 (0.51)	0.79 (0.96)	6.62	0.015	0.442	0.511	0	0.958

Results from repeated measures ANCOVAs controlling for age, sex, education, and employment

*BD*  bipolar disorder, *ED-SD*  emotional distress due to social distancing, *M*  mean, *SD* standard deviation

### Effects of COVID-19 fears/emotional distress to social distancing during the lockdown on psychological symptoms post-lockdown

The regression analyses were ran with selected predictor variables (COVID-19 fears and emotional distress due to social distancing at T1) and control variables (age, sex, education, and employment) using the ENTER method, and the BSI-18 subscales as dependent variables. Results of regression analyses confirmed the hypothesis that COVID-19 fears during the lockdown were associated with psychological symptoms post-lockdown, this was bipolar-specific. In detail, in the BD group, the regression models adjusted for age, sex, education, and employment, which accounted for 59% of the variance (see Table 5), demonstrated that COVID-19 fears predicted somatization (*r* = 0.54, *p* = 0.007; *F*_(6, 19)_ = 3.15, *p* = 0.039, *R*^*2*^ = 0.59). Age was identified as significant confounder in the regression model (*r* = 0.40, *p* = 0.041). No associations between COVID-19 fears and somatization was observed in the control group (*r* = 0.08, *p* = 369; *F*_(6, 19)_ = 0.38, *p* = 0.878, *R*^*2*^ = 0.15). Table 5 lists the regression coefficients and regression results on somatization in both groups. The other regression models to predict anxiety (BD: *F*_(6,19)_ = 1.35, *p* = 0.305, *R*^*2*^ = 0.62; Controls: *F*_(6,19)_ = 1.01, *p* = 0.462, *R*^*2*^ = 0.56), depression (BD: *F*_(6,19)_ = 1.96, *p* = 0.146, *R*^*2*^ = 0.69; Controls: *F*_(6,19)_ = 2.02, *p* = 0.135, *R*^*2*^ = 0.70), and GSI (BD: *F*_(6,19)_ = 1.92, *p* = 0.152, *R*^*2*^ = 0.69; Controls: *F*_(6,19)_ = 1.09, *p* = 0.418, *R*^*2*^ = 0.58) were not significant. Table 6 shows partial correlations (corrected for sex, age, education, and employment) between the regression variables. In BD, associations between emotional distress due to social distancing at T1 and depression at T2 were observed (*r* = 0.63, *p* = 0.009). COVID-19 fears at T1 were related to post-lockdown somatization (*r* = 0.68, *p* = 0.004), anxiety (*r* = 0.53, *p* = 0.035), and GSI (*r* = 0.63, *p* = 0.009). No associations between COVID-19 fears/emotional distress due to social distancing and psychological symptoms within the control group were found.

**Table 5:** Multiple linear regression of COVID-19 fears/emotional distress due to social distancing during lockdown (T1) associated with somatization at post-lockdown (T2)

	Unstandardized coefficients		Standardized coefficients	*t*	*p*	*F *(*p*)	*R*	*R2*	*ΔR2*	*Cohens f2*
	*B*	*SE*	*β*							
Somatization										
BD										
(Constant)						3.15 (0.039*)	0.77	0.59	0.40	1.44
COVID-19 fears	1.36	0.45	0.67	3.00	0.010*					
ED-SD	−0 .38	1.04	− 0.08	− 0.37	0.718					
Age	0.18	.06	0.50	2.75	0.017*					
Sex	− 0.60	2.13	− 0.06	− 0.28	0.783					
Education	0.31	0.73	0.08	0.42	0.680					
Employment	−0 .74	0.79	− 0.18	− 0.94	0.363					
Controls										
(Constant)						0.38 (0.878)	0.38	0.15	− 0.24	0.18
COVID-19 fears	− 0.13	0.25	− 0.18	− 0.53	0.605					
ED-SD	0.82	1.25	0.27	.66	0.520					
Age	0.05	0.07	0.30	.74	0.475					
Sex	− 0.08	1.01	− 0.02	− .08	0.941					
Education	0.46	0.39	0.35	1.17	0.263					
Employment	− 0.00	0.28	− 0.00	− .01	0.994					

*BD*  bipolar disorder, *ED-SD*   emotional distress due to social distancing

*p < 0.05 in bold; multiple linear regression results on somatization with COVID-19 fears and ED-SD as predictors corrected for age, sex, education, and employment

**Table 6:** Partial correlations between COVID-19 fears/emotional distress due to social distancing at T1 and BSI-18 scales at T2 in BD patients and healthy controls

	Somatization		Depression		Anxiety		GSI	
	BD	Controls	BD	Controls	BD	Controls	BD	Controls
COVID-19 fears	0.68**	− 0.06	0.43	0.27	0.53*	− 0.03	0.63**	0.08
ED-SD	0.32	0.12	0.63**	0.14	0.13	0.45	0.43	0.31

*BD*   bipolar disorder, *ED-SD*  emotional distress due to social distancing, *GSI*  global symptom index

**p < 0.01, *p < 0.05 in bold letters; partial correlations were corrected for age, sex, education, and employment

## Discussion

This was the first study to survey individuals diagnosed with BD during the COVID-19 pandemic at two points of measurement—at the peak of the first Austrian lockdown in April 2020 and post-lockdown in May 2020—using a follow-up control-group design. Due to very little empirical data on the impact of the pandemic and lockdown on patients with BD, the aim of this study was to explore psychological consequences of the lockdown during the COVID-19 pandemic in this vulnerable group in order to develop possible strategies for patients to cope with the crisis.

Patients experienced social distancing during the first Austrian lockdown in April 2020 as much more burdensome than controls. They reported more emotional distress due to social distancing (including feelings of loneliness, boredom, frustration, hopelessness, and anxiety). Although the regression model revealed no effect, partial correlations in BD showed a relationship between emotional distress due to social distancing and depression scores at post-lockdown. In addition, patients with BD reported high values in depression, anxiety, and psychological distress (measured with GSI) during lockdown. This was in line with previous studies in samples with severe mental disorders during the COVID-19 pandemic in 2020 (Carmassi et al. 2020; González-Blanco et al. 2020; Solé et al. 2021). In comparison to healthy controls, individuals with severe mental illness reported less use of coping strategies, such as having a routine, social interactions, and a healthy lifestyle (Solé et al. 2021).

We suppose that this first lockdown in Austria may have led to high insecurity and anxiety with affective dynamics in patients with BD compared to controls. According to the literature, the ongoing COVID-19 pandemic is suggested to promote worries about health, personal and financial loss, uncertainties, anger, confusion, frustration, boredom, decrease in social contacts, isolation, loss in daily routine, stigma, emotional distress, and exacerbation of psychological symptoms in otherwise healthy individuals (Barzilay et al. 2020; Brooks et al. 2020; Fatke et al. 2020; Lieberman et al. 2020; Pérez-Fuentes et al. 2020; Pfefferbaum and North 2020; Tull et al. 2020). Certain aspects of the pandemic could affect patients with BD, especially concerning the risk of relapse or disruption in biological and social rhythm (Rajkumar 2020). Studies have shown that traits related to emotional instability and anxiety are generally elevated in bipolar spectrum disorder (Evans et al. 2005; Greenwood et al. 2012; Wagner-Skacel et al. 2020). It is suggested that individuals with BD are less securer and more anxious than healthy people in normal situation and that this it getting more severe under extreme situation. Individuals with BD are prone to react to stress more extensively (Stefana et al. 2020), and this is in line with results of past epidemics and natural disasters (Esterwood and Saeed 2020) as well as recent longitudinal studies during the COVID-19 pandemic in individuals with affective disorders (Frank et al. 2020; Pan et al. 2020). The real development of COVID-19 pandemic effects on psychological conditions in patients with BD as a response to the crisis will be objects of further investigations.

There is a link between personality structure and affective dynamics, including depressive, anxiety, and somatization symptoms in BD. An impairment of personality functioning including difficulties in interpersonal relations as well as self-regulation in individuals with BD leads to more psychological distress (Wagner-Skacel et al. 2020). Additionally, we know that patients with BD usually engage in ineffective coping strategies to address stressful situations (Bender and Alloy 2011). These patients may need more stabilization in the affective regulation during the COVID-19 pandemic.

Using a 2 × 2 design, it was evident that patients with BD got closer to healthy controls from April to May in somatization, whereas healthy controls showed no significant change, rather decline, in somatization. This divided reaction of patients and controls may be caused by a rising frustration and strain over time in the control population. For people without a history of mental illness, this period of chronic emotional stress und uncertainty may have been a first-time experience. Possibly, their natural resilience may have hit its limit in May, resulting in a deterioration of their psychological condition. We suggest that patients with BD might be more familiar with such episodes of high stress. In general, we assume that healthy individuals maintain a more active social life than psychiatric individuals, wherefore changes in the social structure could strain them highly.

The decreasing trends in somatization observed in May 2020 in patients might be interpreted as depending on easing of strict lockdown. The second point of measurement was after the lockdown, along with reopening of schools, nurseries, kindergartens, restaurants/bars, hotels, recreational centers, shops, theaters, etc., when social life started to rise. We suppose that the easing of lockdown measures and thus the revival of social contact, reconstitution of psychiatric treatment, and return to “normal life” correlated with the decrease in psychological symptoms, especially somatization symptoms. Somatization is suggested as an indicator for psychological distress in the form of somatic symptoms. A recent meta-analysis revealed that medically unexplained somatic symptoms are highly prevalent among persons with BD at a rate nearly double that of the general population (Edgcomb et al. 2016). The same study group investigated predictors and outcomes of somatization in patients with BD. Somatic symptoms were independently associated with disease severity, defined as earlier age of first seeking psychiatric help and first psychiatric hospitalization, greater probability of attempting suicide, and rapid cycling course of disease (Edgcomb and Kerner 2018). Studies on somatization in patients with BD have been limited to anxiety subscale scores of somatization, establishing that anxiety disorders occur more frequently in persons with BD than in the general population (Pavlova et al. 2015). Research on the psychological reactions to previous epidemics suggest various vulnerability factors for anxiety and fears associated with an infectious virus, such as individual differences in intolerance of uncertainty and perceived vulnerability to disease (Esterwood and Saeed 2020; Taylor et al. 2004). In this study, COVID-19 fears could predict later somatization in the BD group. Somatization refers to psychological stress caused by the perception of physical dysfunctions. The somatization items of the BSI-18 focus on body symptoms with strong autonomous mediation (Franke et al. 2017). Subjective perception, thoughts, emotions, and behaviours associated with the individual somatic status are sometimes clinically more important than a medical diagnosis. In this context, it is very important to identify anxiety and COVID-19 fears in the BD group, but also in the general population, and to find evidence-based ways of addressing these issues for future outbreaks of infection.

Although we suggest that the change in symptoms is related to the easing of restrictions, some patients with BD might adapt and perhaps benefit from the complete stop of social obligations, resulting in decreased somatization scores. Other studies found a decrease of symptoms during the course of the COVID-19 pandemic too (Orhan et al. 2020). According to our results, we see a chance for patients individually, as some might realize that others (normally nonimpaired fellow human beings) are also afraid of the consequences of the COVID-19 pandemic, e.g., fear to contract the coronavirus or to infecting others with the coronavirus.

BD has high comorbidity with obesity; metabolic disorders including diabetes mellitus, coronary heart disease, and obstructive pulmonary disease; and smoking and substance abuse (Staudt Hansen et al. 2018; McInytre et al. 2007; Vancampfort et al. 2013; Vancampfort et al. 2016; Zareifopoulos et al. 2018). These related somatic illnesses compromise immune functioning and heighten the risk for SARS-CoV-2 infection per se as well as a severe course if one is infected (Stefana et al. 2020; Yao et al. 2020). Based on this, it is surprising that our results show that patients with BD are not more afraid to contract the coronavirus than healthy controls. At both time points, patients and controls did not differ in COVID-19 fears, including general concerns and fears about the coronavirus, fear of contracting the coronavirus, and fear of infecting others with the coronavirus. We suppose that patients with BD are not always aware of the high somatic risk factors accompanying their psychiatric disease. Thus, integrating more psychoeducational intervention programs, possibly smartphone based, related to increased somatic risk factors and SARS-CoV-2 infection would be beneficial in the treatment of BD. Otherwise, in this context it must be mentioned that lithium has shown direct antiviral effects, which might possibly protect individuals with BD against SARS-CoV-2 infection (Murru et al. 2020; Nowak and Walkowiak 2020). Stefana et al. (2020) indicated that social stigma flares when societies are under stress. Particularly at risk for stigmatization are vulnerable groups or those considered as “different”. They suggested that, as BD is already prone to stigmatization, people with BD will undoubtedly take a second hit when they contract COVID-19 (Stefana et al. 2020).

### Limitations

Several limitations should be considered when interpreting the current results. First, we only assessed patients with BD who were former study participants and who were motivated to participate in an online survey. Therefore, results may not be transferable to patients with BD who have not yet received therapy. We have not assessed euthymia by external rating criteria and do not know the affective status of the patients. However, affective state was monitored using self-ratings (BDI-2, ASRM). Additionally, the gender distribution in the control sample was unequal, there were more male patients than healthy controls. In addition, compared to controls, more patients were retired or in rehabilitation or had completed apprenticeship or A-Levels, which could have influenced the results. Since adverse course of COVID-19 is strongly associated with age, it cannot be ruled out, that differences in symptoms may be at least partially due to the differences in age (which has been controlled in all analyses).

Second, all data were self-reported and, thus, may potentially be biased. Although we assessed COVID-19 fears and emotional responses in addition to psychological scales, it cannot be excluded that at least some symptom worsening was not directly attributable to the COVID-19 pandemic. In addition, we do not know whether the participants were being negative tested for COVID-19 or have not being tested, as this discrimination was not assessed at the beginning of the pandemic. Third, COVID-19 fears and emotional distress due to social distancing were self-conducted variables and not measured by validated, standardized questionnaires. Fourth, we only included patients with complete data at both time points, and therefore the sample was relatively small and may be too small to be of much importance. Fifth, the observation period was at the beginning of the crisis and relatively short in order to display the social effects of the pandemic such as job loss or isolation. In this respect, follow-up investigations of this study are of special interest. Sixth, we have no information on BSI-18 scores in our sample before the outbreak of the COVID-19 pandemic or prior to lockdown.

### Clinical implications

There is a need for adequate and necessary attention to people with BD in the COVID-19 pandemic and to develop specific health care interventions and treatment approaches for this particular vulnerable group during the crisis. Given the high scores in scales of depression, anxiety, and somatization during the lockdown and the finding that COVID-19 fears during the lockdown predict later symptoms in individuals with BD, interventions aimed at helping patients cope with fears and symptoms might help manage their condition. In this context, telemedicine, which obviates the risk of virus transmission inherent in face-to-face therapy, offers a great potential for delivering treatment during the COVID-19 pandemic (Torous and Wykes 2020; Wind et al. 2020; Zhou et al. 2020). Our findings show that videoconference therapy was used by a small part, only 22% of patients. However, we do not know if there was too little offer or demand. In any case, we believe that online treatment (especially during lockdown) has to be expanded significantly in the future, and it would be worthwhile to provide online or smartphone-based psychological interventions (e.g., cognitive behavioral therapy and mindfulness-based therapy) (Szentagotai and David 2009; Williams et al. 2008). In addition, patients with BD need more information about risk factors (disruption of daily rhythms, social isolation, somatic comorbidities) and their vulnerability to stress in order to protect themselves better during any time of great social disruption, whether caused by pandemic, environmental disaster, or whatnot. Specific psychosocial and psychological interventions during a pandemic could be relaxation methods and maintenance of daily rhythms. The self-awareness of patients with high somatization symptoms could be improved using strategies and skills for emotion regulation, engagement in stress-reducing activities, the regulation of relationships, and healthy lifestyle.

Much like Stefana et al. (2020) and Youngstrom et al. (2020), we are convinced that every crisis could be an opportunity by learning and rethinking and therefore gaining a more in-depth understanding of BD patients’ special needs during the pandemic.

### Conclusion

Our findings summarize that the COVID-19 pandemic and especially the lockdown measures greatly challenged patients with BD. At the peak of the Austrian lockdown in April 2020, psychological symptoms were observed in patients with BD; somatization decreased in patients from April to May 2020 along with easing of restrictions, in contrast to controls, and we conclude that this was dependent on lockdown measures. Accordingly, we want to emphasize that fears of contracting the coronavirus or fears of infecting others (so called COVID-19 fears) during the lockdown could predict later symptoms of somatization and that these results were bipolar specific. We therefore propose that patients with BD need more information about potential lockdown effects on their psychological well-being and about strategies for stress reduction and dealing with fears during periods of reduced psychosocial care. Patients with a higher vulnerability, including less resilience, maladaptive coping strategies, higher psychological distress, more severe course of disease, and more somatic comorbidities, have to be identified earlier and need frequent clinical contacts with a more active role of the therapist or doctor during times of crisis. Follow-up studies to estimate the long-term effects of the COVID-19 pandemic in individuals with BD are highly needed.

## Data Availability

The datasets generated and or analysed during the current study are available from the corresponding author on reasonable request.

## Abbreviations

SARS-CoV-2: Severe acute respiratory syndrome coronavirus-2
COVID-19: Coronavirus disease
BD: Bipolar disorder
SCID-I: Structured Clinical Interview for DSM-IV
BSI-18: Brief Symptom Inventory-18
GSI: Global severity index
BDI-2: Beck depression inventory
ASRM: Altman self rating mania scale
MANCOVA: Multivariate covariance analyses
SPSS: Statistical Package for Social Sciences
ANCOVA: Analysis of covariance
